# Revised taxonomy of eastern North Pacific killer whales (*Orcinus orca*): Bigg’s and resident ecotypes deserve species status

**DOI:** 10.1098/rsos.231368

**Published:** 2024-03-27

**Authors:** Phillip A. Morin, Morgan L. McCarthy, Charissa W. Fung, John W. Durban, Kim M. Parsons, William F. Perrin, Barbara L. Taylor, Thomas A. Jefferson, Frederick I. Archer

**Affiliations:** ^1^ Southwest Fisheries Science Center, National Marine Fisheries Service, NOAA, La Jolla, CA 92037, USA; ^2^ University of British Columbia, Vancouver, British Columbia V6T 1Z4, Canada; ^3^ Marine Mammal Institute, Oregon State University, Newport, OR 97365, USA; ^4^ Northwest Fisheries Science Center, National Marine Fisheries Service, NOAA, Seattle, WA 98112, USA

**Keywords:** Cetacea, odontocete, speciation

## Abstract

Killer whales (*Orcinus orca*) are currently recognized as a single ecologically and morphologically diverse, globally distributed species. Multiple morphotypes or ecotypes have been described, often associated with feeding specialization, and several studies have suggested taxonomic revision to include multiple subspecies or species in the genus. We review the ecological, morphological and genetic data for the well-studied ‘resident’ and Bigg’s (aka ‘transient’) ecotypes in the eastern North Pacific and use quantitative taxonomic guidelines and standards to determine whether the taxonomic status of these killer whale ecotypes should be revised. Our review and new analyses indicate that species-level status is justified in both cases, and we conclude that eastern North Pacific Bigg’s killer whales should be recognized as *Orcinus rectipinnus* (Cope in Scammon, 1869) and resident killer whales should be recognized as *Orcinus ater* (Cope in Scammon, 1869).

## 1. Introduction

Killer whales (*Orcinus orca,* also called orcas) are apex predators found in all the world’s oceans [1]. Up to 23 species and four subspecies of killer whales have been named in the literature [2], many based on the skull morphology of single specimens. These taxa have been synonymized into the monospecific genus *Orcinus* [3,4], and currently, a single species, *O. orca*, is recognized globally [5].

A suite of studies on morphological, behavioural, acoustic, genetic and other data have indicated that the currently recognized species may comprise multiple unrecognized taxa (e.g. [6–9]). This was highlighted by a review of killer whale differentiation as a case study at a workshop on the shortcomings of cetacean taxonomy in relation to the needs of conservation and management [10]. The participants of the workshop determined that while it was likely there were multiple subspecies or species of killer whales globally, the data available at the time of the review (30 April to 2 May 2004) were insufficient to allow conclusions for taxonomic revision.

Regional variation in a number of characteristics has led to the recognition of several forms of killer whales, which are often referred to as ‘ecotypes’ [6,7,11–17]. These ecotypes are known to vary in body size [16,18–20], colour patterning [6,16,21–23], social structure [24–28], vocalization pattern [28–31] and foraging strategies [6,11,32–39].

**Figure 1.**
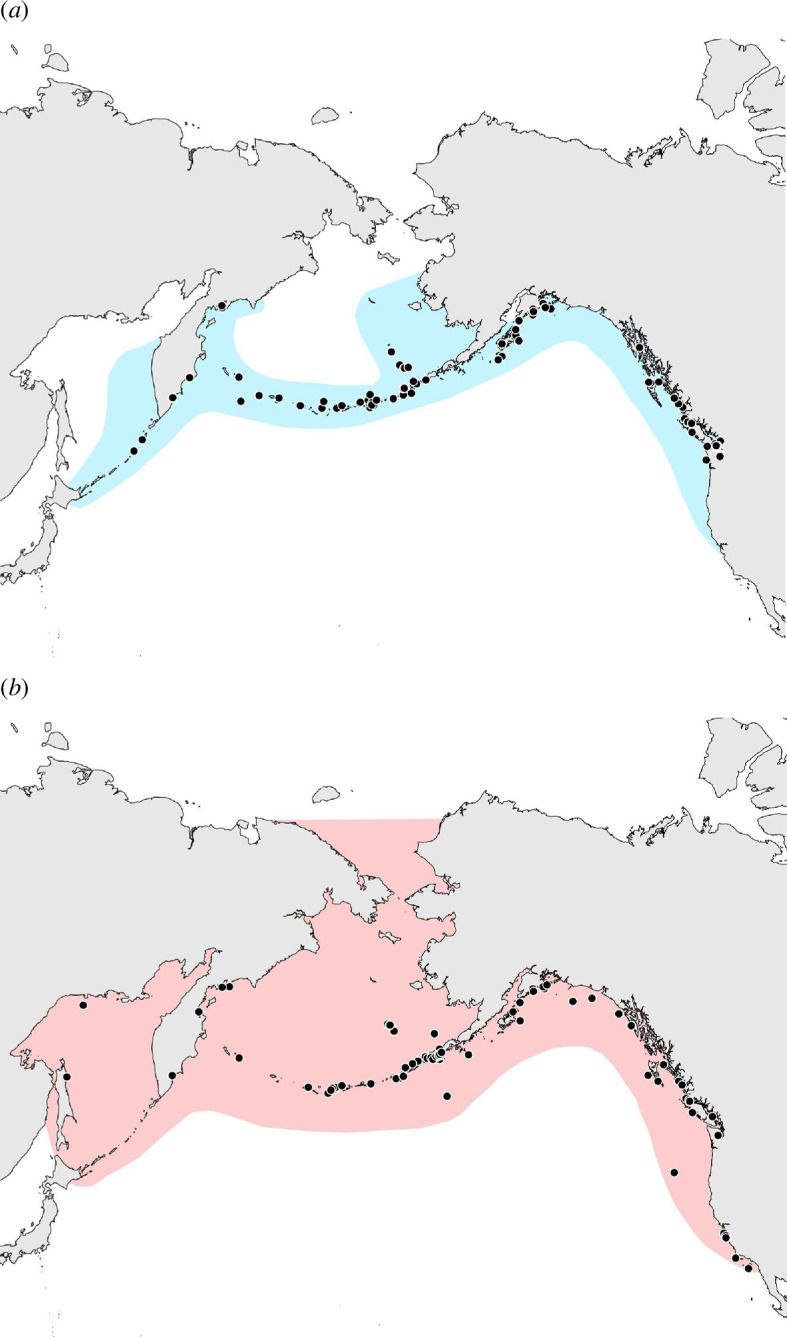
Expected range maps for (*a*) resident and (*b*) Bigg’s killer whales, including locations of samples used for mitogenome analysis (figure 5*a*, resident *n* = 106, Bigg’s *n* = 93) [15]. Distribution ranges have been inferred based on published identifications of individuals that are identified by ecotype [48–53]. Sample distributions cover the known ranges of both ecotypes, with the exception of residents of Oregon and northern California, and both ecotypes off northern Japan (Hokkaido) in the western Pacific [48,54]. Sample maps for microsatellite data are in electronic supplementary material, figure S2.

In the North Pacific, three killer whale ecotypes have been described. Bigg’s killer whales (aka ‘transients’) are mostly observed on the continental shelf in temperate to Arctic waters, though their distribution beyond the shelf is not well documented, and they specialize in eating marine mammals [25,40–42]. So-called resident killer whales (hereafter referred to as residents) are primarily coastal and are found mostly in waters north of central California in the eastern Pacific (with seasonal visits to northern or central California; e.g. [43]) and as far west as Russian coastal waters in the western Pacific (figure 1). Residents are known to specialize in eating fish, especially salmon [39,41,42,44,45]. Offshore killer whales are found primarily in waters off the continental shelf and therefore are less known, but they appear to prey on a variety of fish, especially elasmobranchs [33,41,46,47]. For all three ecotypes, the extent of their range in the western Pacific is unknown. At latitudes below ~34° N off the coasts of California and Mexico, and in the eastern tropical Pacific (ETP), ecotypes are not well defined. It is unclear whether there are ecological or reproductive barriers between killer whales in these lower latitudes and the more northerly Bigg’s or offshore ecotypes. Residents appear to be geographically limited to higher latitudes, where they are unlikely to contact the lower-latitude populations given current known ranges.

Previous analyses of mitochondrial genomes [7,15,55], microsatellites [56], single-nucleotide polymorphisms (SNPs) [15,57,58] and nuclear sequences [13,59,60] from killer whales indicated that several killer whale ecotypes represent genetically distinct groups with no evidence of contemporary gene flow, suggesting the need for taxonomic re-evaluation. Here, we focus on the taxonomy of the Bigg’s and resident ecotypes in the North Pacific, for which extensive data on different characteristics have been published, allowing taxonomic inference. These two ecotypes have previously been proposed to represent separate unnamed subspecies [5], following a status review in 2004 [61]. Although there is also evidence that residents and Bigg’s are distinct from other types of killer whales [15,57,59,62], comparable data from most other ecotypes and regions are limited, precluding comprehensive comparisons. It is important to recognize that a lack of complete information for a global taxonomy for the genus is not a sufficient reason to resist taxonomic classification where sufficient data permit the testing of taxonomic hypotheses [63]. Indeed, taxonomy is the science of delimiting species (and other taxonomic groups) and should continue to progress through research and understanding of biodiversity and classification as with any other hypothesis-driven scientific endeavour [64].

We summarize and evaluate available data relevant for the evaluation of the taxonomic status of the resident and Bigg’s ecotypes in the context of explicit subspecies and species concepts. Historically, subspecies have been described as geographically separate and diagnosably distinct breeding populations [65,66], while the literature on species concepts is extensive (e.g. [67–70]). Here, we follow the definitions from Taylor *et al*. [63] for species defined as ‘a separately evolving lineage composed of a population or collection of populations’ and for subspecies as ‘a population, or collection of populations that appears to be a separately evolving lineage with discontinuities resulting from geography, ecological specialization or other forces that restrict gene flow to the point that the population or collection of populations is diagnosably distinct’.

Following guidelines for subspecies and species delimitation provided by Taylor *et al*. [71], we evaluate multiple lines of evidence to determine whether resident and Bigg’s ecotypes meet the suggested criteria for subspecies or species, relative to each other and to the remaining nominate species where data are available. We consider quantitative morphological and genetic evidence (diagnosability, reciprocal monophyly, fixed differences in mitochondrial and nuclear loci) as strong evidence for species, while qualitative (statistical) and quantitative data types (mitochondrial DNA (mtDNA) net divergence) are considered strong evidence for subspecies [71,72]. To determine whether the two ecotypes satisfy the criteria for taxonomic reclassification as subspecies or species, we specifically address five questions, the first four of which inform evaluations of taxonomic level:

**Distinctness:** Are the ecotypes genetically distinct?

**Differentiation:** Is there evidence of ongoing male-mediated gene flow?

**Diagnosability:** Are individuals diagnosable based on genetic and/or morphological characteristics?

**Divergence:** Is the divergence between the ecotypes sufficiently large^1^ to indicate separately evolving lineages?

**Taxonomic level:** If the ecotypes should be considered different taxa, are they subspecies or species? Specifically, do they meet the specified criteria for diagnosability, and is there sufficient evidence to distinguish between the taxa ‘appearing’ to be separately evolving lineages and conclude that they are separately evolving lineages? Threshold values of net nucleotide divergence (*d*_A_) have been proposed as a genetic proxy for the latter, though multiple independent lines of evidence, such as morphological data or evidence for adaptive genetic divergence, are considered to be important for determining species status, when available [71,73,74].

## 2. Review of lines of evidence

### 2.1. Ecology and behaviour

#### 2.1.1. Distribution and social structure

Both Bigg’s and resident killer whales are found across the North Pacific Ocean basin and overlap greatly in coastal regions where most research has taken place (figure 1). As such, they have the potential to interbreed. However, in over 50 years of field study, the ecotypes have never been seen to associate, and there is observational evidence that the two ecotypes exhibit active avoidance of each other, and sometimes act aggressively [11,75,76], leading to the suggestion that they are reproductively isolated, possibly owing to cultural divergence [27,75].

Killer whales are highly social, but these ecotypes differ in average group size, dispersal, acoustic communication and social organization. Residents are unique in having large, stable groups with negligible permanent dispersal from their natal group [25,77–80], though groups have been observed to permanently split along matrilines [81]. Bigg’s are typically found in smaller groups of maternally related individuals, with permanent dispersal of females following the birth of their first calf, while there is evidence of a high degree of male philopatry [24,76,79,82].

Behavioural avoidance and a lack of interbreeding between ecotypes in sympatry address the question of distinctness and support species status under the biological species concept.

#### 2.1.2. Ecology and feeding

Dietary specialization is a common distinguishing characteristic of killer whale ecotypes [6,11,12,18,33,36,37,40–42,44,76,83]. Prey specialization was the primary evidence initially used to identify the fish-eating resident and mammal-eating Bigg’s ecotypes in the eastern North Pacific [11,34,42]. Multiple methods have been used to determine dietary specialization and seasonal diet changes for residents and Bigg’s, including behavioural observations and examination of prey remains [11,42], molecular prey species identification from faeces [45,84], stable isotopes [41,47,85,86], pollutants [41,47,87,88] and fatty acids from skin and blubber biopsies [41,47]. Because of their higher trophic level, the marine mammal-eating Bigg’s exhibit higher δ15 N and δ13 C isotope levels, lower proportions of omega-3 long-chain mono-unsaturated and poly-unsaturated fatty acids (evidence of fish consumption) and higher levels of persistent organic pollutants than fish-eating residents [89].

Diversifying selection associated with foraging strategies can drive divergent evolution in behavioural traits such as habitat use, seasonal movements and foraging behaviours and has been proposed to have played a major role in the social isolation and genetic divergence of killer whale ecotypes (reviewed in [83]). Behaviours associated with feeding specialization include large differences in foraging group size, with residents’ average group size of 18, often increasing temporarily with high prey density [79]. Foraging for marine mammals by Bigg’s killer whales is associated with small groups (range 2–6) hunting by stealth (see §2.1.3). Residents’ seasonal movements have been linked to salmon species aggregations ([42, 44], reviewed in [83,90,91]), while Bigg’s occurrence is relatively uniform, with frequent travel and seasonal peaks associated with the pupping season of harbour seals and the migration of grey whales [40,75,76,92].

The multiple methods of studying feeding specialization all strongly indicate that there is no overlap in primary diet between residents and Bigg’s, and many behavioural, acoustic and morphological characteristics (see below) have developed in the ecotypes, showing distinctness, differentiation and diagnosability.

#### 2.1.3. Acoustics

Killer whales use a variety of acoustic call types to communicate and forage for food, and ecotypes can be identified acoustically by their group-specific pulsed calls [30,35,93]. While not diagnostic, several acoustic characteristics differ significantly between ecotypes. Bigg’s whistles differ from resident whistles in duration, end frequency and maximum frequency [29,94–96]. Resident sonar click trains are produced on average 6–27 times more often and are twice as long as in Bigg’s whales, though the frequency of vocalization is context dependent [29,76,97]. Bigg’s are typically silent while foraging, while residents are highly vocal, communicating with each other and using echolocation to detect prey [29,76,98–100].

Acoustic differences show divergence between the ecotypes, relevant to differentiation associated with culture and feeding ecology.

### 2.2. Morphology

**Figure 2.**
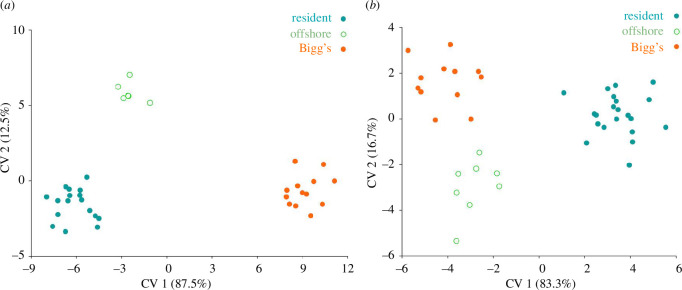
Canonical variate 1 and 2 plots for cranial shape features that distinguish among ecotypes for (*a*) skull morphology (resident (*n* = 17), Bigg’s (*n* = 13) and offshore (*n* = 6)) and (*b*) dentary bone morphology (resident (*n* = 21), Bigg’s (*n* = 12) and offshore (*n* = 8) specimens) (reprinted from [103]).

Resident and Bigg’s ecotypes can be reliably distinguished by experienced observers at sea and from field observations, photographs and video, based on external characteristics such as eye patch and saddle patch pigmentation, the size and shape of the dorsal fin and differences in body size [22,23,27,34,85,101,102]. The Fourier analysis allowed quantitative discrimination among ecotypes based on the dorsal fin and eye patch [23], but these characteristics vary substantially among individuals and by sex. Morphometric data have been difficult to obtain, but skull morphology based on several characters reveals diagnostic shape differences between the North Pacific ecotypes (figure 2) [103]. Recent photogrammetry data from free-swimming sympatric resident and Bigg’s killer whales in the eastern North Pacific have also shown significant differences in body length and condition, with Bigg’s of both sexes longer and more robust than residents [20].

In cetaceans, diagnosable morphological differences arise from substantial reproductive isolation [104–106]. Multivariate analysis of the skull and jaw structures of resident and Bigg’s ecotypes in particular results in diagnostic differences (figure 2) that correspond with dietary specializations, e.g. deeper curvature of the jaw, pronounced convex ventral edge in Bigg’s, potentially correlated with the demands of biting and gripping large prey [103]. These morphological differences suggest distinctness, differentiation and diagnosability. More morphological and behavioural data are still needed from the offshore ecotype, but preliminary data indicated diagnostic differences among all three ecotypes (figure 2).

### 2.3. Molecular genetics

Early genetic analyses of killer whales were based on the standard markers of the time: mitochondrial control region sequences and nuclear microsatellite genotypes. With their extremely low genetic diversity and recent divergence, taxonomic interpretations of the results of these analyses were inconclusive [8,107–112]. Larger numbers of variable markers helped to clarify relatedness, population structure and ecotype differences [56,78], but it was not until advancements in high-throughput genome sequencing methods, which allowed for population-level sequencing of complete mitochondrial genomes (mitogenomes) and genome-wide nuclear variants, that patterns of divergence on an evolutionary scale among ecotypes and global populations began to resolve [7,9,13–15,55,58,113].

#### 2.3.1. Differentiation and divergence metrics

Based on analysis of patterns of divergence and diagnosability for a variety of recognized cetacean populations, subspecies and species, Taylor *et al.* [71] published a set of guidelines for subspecies and species designation when only mtDNA control region sequence data were available. Resident and Bigg’s killer whale ecotypes meet the criteria for subspecies based on these guidelines for mtDNA control region sequences alone (500 bp, *d*_A_ > 0.004). They also meet subspecies thresholds based on complete mitochondrial genomes (*d*_A_ > 0.0006) [114], with 100% diagnosability (table 1). In neither dataset do they meet the higher *d*_A_ threshold for species (*d*_A_ > 0.02 for the control region and >0.008 for the mitogenome). In the Taylor *et al.* [71] study, the authors note that guidelines should not be applied rigidly and that exceptions are expected, especially under divergent selection and/or when population sizes are very small and/or culturally driven niche partitioning may lead to more rapid evolution and high diagnosability despite low mitochondrial divergence [114]. All of these conditions are likely to apply to North Pacific resident and Bigg’s killer whale ecotypes.

**Table 1.** Measures of differentiation, divergence and diagnosability based on nuclear microsatellite and SNP data. All frequency-based metrics (*F*_ST_, *F’*_ST_, *G’*_ST_) were significantly different from zero at *p* < 0.05.

divergence metric		reference
*d*_A_ (CR)	0.00746^a^	[114]
*d*_A_ (mitogenome)	0.00391^b^	[114]
*F*_ST_ (microsatellite)	0.21–0.23	[56,85]
*F’*_ST_ (microsatellite)	0.47	[56]
*G’*_ST_ (microsatellite)	0.28	[56]
*F*_ST_ (SNP)	0.28	[15]
*F*_ST_ (RADseq, neutral)^c^	0.27	[58]
*F*_ST_ (RADseq, selected)^c^	0.67	[58]
*F*_ST_ (genomes)	0.32	[9] (electronic supplementary material, table S2)
diagnosability (CR)	100%	[114]
diagnosability (mitogenome)	100%	[114]
fixed differences (mitogenome)	57	[7]
fixed differences (3281 nuclear SNPs)	2–7	[58]
fixed differences (6 435 100 nuclear SNPs)	6361^d^	[113]

^a^ 95% confidence interval: 0.00727–0.00772.

^b^ 95% confidence interval: 0.00388–0.00393.

^c^ Alaska transients (Bigg’s) versus Alaska residents.

^d^ Based on analysis of raw SNP data from Kardos *et al*. [113].

**Figure 3.**
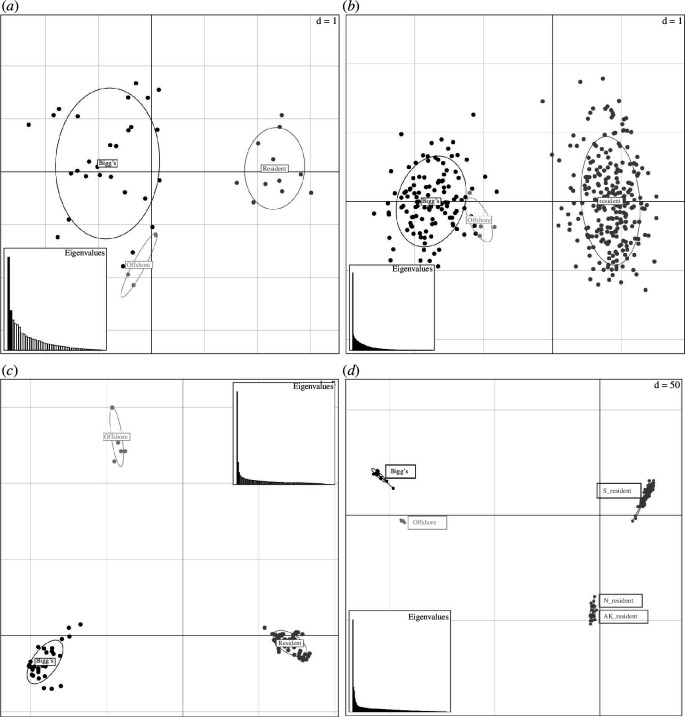
PCA plot of first two principal components based on (*a*) 88 SNPs: offshore (*n* = 3), resident (*n* = 11), Bigg’s (*n* = 30) from data in Morin *et al*. [15]; (*b*) 26 microsatellites: offshore (*n* = 5), resident (*n* = 250), Bigg’s (*n* = 116) (samples genotyped at ≥20 loci) [56]; unpublished); (*c*) 3678 RADseq SNPs: offshore (*n* = 7), resident (*n* = 52) and Bigg’s (*n* = 37) populations [57,62]; (*d*) 1 00 000 (subset from 6 371 282) SNPs from 147 high-coverage genomes of offshore (*n* = 7), Bigg’s (*n* = 14) and resident (*n* = 126) samples from multiple geographically and behaviourally defined subpopulations (Alaska, northern and southern resident populations) (based on subset of SNP genotype data from [113]. See Supplementary Materials for methods and data set information.

**Figure 4.**
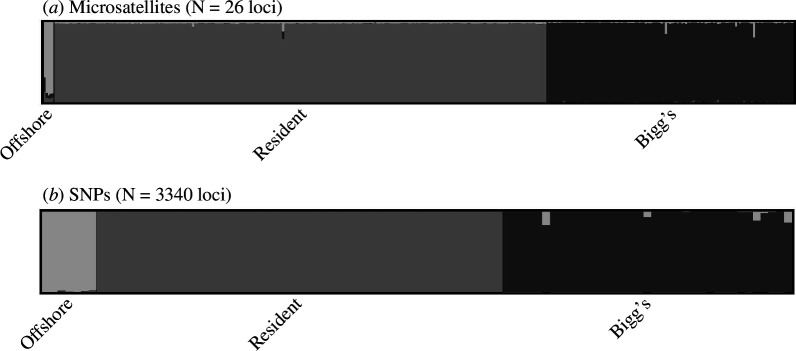
Structure assignment probability plots for *K* = 3 groups from (*a*) 26 microsatellites: offshore (*n* = 5), resident (*n* = 250), Bigg’s (*n* = 116) samples genotyped at ≥ 20 loci) (56; unpublished); (*b*) 3340 RADseq SNPs (polymorphic in sample set): offshore (*n* = 7), resident (*n* = 52) and Bigg’s (*n* = 37) populations [57,62]. Vertical bars represent the individual assignment probability for each group inferred by Structure (groups identified by shading), with samples sorted by *a priori* ecotype assignment. See electronic supplementary material for methods and data set information.

Other standard measures of genetic differentiation are less indicative of taxonomic status, especially at the population and subspecies levels [115], although they are correlated and can provide evidence of a lack of current significant levels of gene flow. For residents and Bigg’s, frequency-based measures of divergence (*F*_ST_, *F*′_ST_, *G*′_ST_) based on nuclear microsatellites and SNPs from several studies all showed relatively large and significant differentiation (table 1). Other methods (e.g. principal components analysis (PCA), structure program analysis) based on range-wide sample sets (figure 1) indicated near-perfect assignment of individuals to ecotypes based on genetic data alone (figure 3; see electronic supplementary material for methods). The program structure [116] uses a Bayesian clustering approach to assign samples to groups based on estimated allele frequencies for populations in Hardy–Weinberg equilibrium. As such, the high probability of assignment of all individuals to populations correlated with ecotype assignment (based on independent data) provides strong evidence of genetically differentiated populations (figure 4; see electronic supplementary material for methods), though the sample size for the offshore ecotype remains low, limiting confidence in assignments based on these methods.

Initial estimates of gene flow suggested limited interbreeding between ecotypes based on a small number of microsatellite loci [111]. Subsequent studies using larger numbers of microsatellites, SNPs and genome-wide markers for paternity, relatedness and population studies have found no evidence of ongoing gene flow between ecotypes [56,58–60,78,117], though genomic analyses indicate historical or episodic gene flow between residents and Bigg’s, possibly through intermediary populations such as the offshore ecotype or ETP populations [58,59].

The combined results from analyses of both mtDNA and nuclear DNA (nuDNA) provide evidence of distinctness, differentiation, diagnosability and divergence.

#### 2.3.2. Phylogenetics

**Figure 5.**
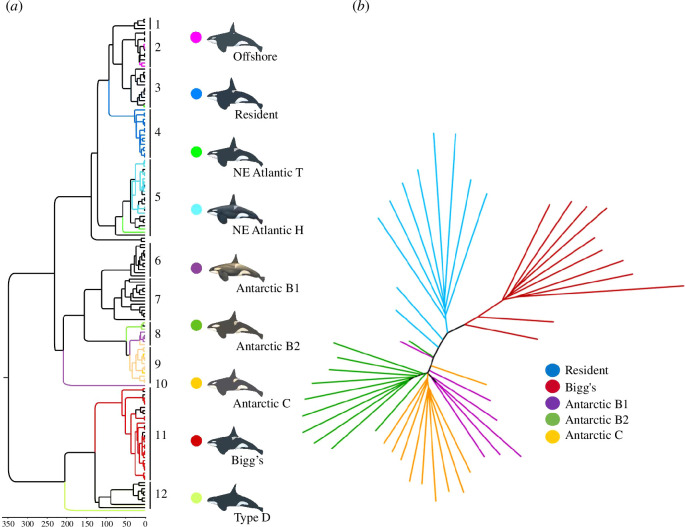
Global phylogenetic trees of killer whales based on (*a*) haplotypes from 452 mitogenomes and (*b*) 49 nuclear genome sequences. Reprinted with permission from Morin *et al*. [15] (figure 2; by permission from John Wiley & Sons, licence 5458310335802) and [9] (electronic supplementary material, figure S3*b*, by permission from Andrew D. Foote). Black branches in (*a*) lead to haplotypes that are from animals that have not been identified to ecotype (see electronic supplementary material, table S1 from [15]).

For the resident and Bigg’s ecotypes, mitogenome phylogenetics showed a surprisingly deep divergence, with complete reciprocal monophyly between haplotypes of the two ecotypes, in different major branches of the global phylogeny (figure 5*a*) [7,15]. Estimates of divergence time between resident and Bigg’s ecotypes ranged from approximately 700 000–350 000 years [7,15], though gene flow among ecotypes likely occurred episodically or via other ecotypes or populations [13,59,62,118], leading to non-conforming phylogenetic trees from different datasets (figure 5; see also Kardos *et al*. [113] and Moura *et al*. [57]), and different interpretations for timing of divergence and gene flow [57,58,62,119].

The mitochondrial and nuclear phylogeographic results provide evidence for distinctness (no overlapping haplotypes), differentiation (clade support and concordance with ecotypes), diagnosability (fixed differences, reciprocal monophyly) and divergence (divergence time).

#### 2.3.3. Demographic histories

The long-term historical demography of killer whales has been estimated from whole-genome sequences using pairwise sequentially Markovian coalescent (PSMC), which infers changes in effective population size (*N*_e_) over evolutionary time (~10 000–1 Myr ago; e.g. [120]). Plots of population sizes for killer whales fall into several patterns over the past ~10–30 kyr, before and during the Last Glacial Maximum, with resident and offshore ecotype populations declining, while Bigg’s population size remained relatively stable [60]. Additionally, PSMC analysis of pseudo-diploids (*in silico* constructs comprised of haploid sequences from two individuals) can be used to determine the approximate time that populations became demographically independent, signalling the end of gene flow. Pseudo-diploid analysis of the X-chromosomes indicates that residents and Bigg’s began to diverge from the ancestral population at different times, with Bigg’s diverging between 200 000 and 300 000 years ago, while residents began to diverge approximately 100 000 years ago [59]. Changes in effective population size have been inferred based on SNPs across the genomes of resident and Bigg’s ecotypes, suggesting that the Bigg’s population remained relatively large (*N*_e_ = 5.5k–6k) and stable until approximately 10 kyr ago, while residents *N*_e_ declined from about 6k to 600 between 40 and 10 kyr ago [60]. More recent demographic change inferred from SNP linkage disequilibrium (LD) indicates that the *N*_e_’s for both ecotypes have declined to <100 within the last ~750 years [113].

Together, the PSMC and LD analyses support divergence and nuclear genome differentiation over several hundred thousand years and continued demographic independence of residents and Bigg’s killer whales in deep (>10 kyr) and recent (10 kyr to present) time periods. Analyses of nuclear and mitochondrial genome samples from residents and Bigg’s (above) indicate that the demographic independence continues to the present day, with no inference of recent or ongoing gene flow.

#### 2.3.4. Functional genomics

Reproductive proteins such as PKDREJ, in which there were two fixed non-synonymous substitutions derived from the resident ecotype, are known to diverge rapidly across taxa and are potential candidates for post-zygotic isolation [13]. Genes encoding proteins associated with dietary variation were also shown to be under divergent selection, including genes associated with the regulation of methionine metabolism in mammal-eating ecotypes and associated with carboxylic ester hydrolase activity in residents [13]. Despite low-coverage genome sequencing, Foote *et al*. [13] detected dozens of significant non-synonymous allele changes in genes, indicating evidence of recent selection in each ecotype (electronic supplementary material, table S10 from [13]).

Genes under strong divergent selection in ecotypes require substantial reproductive isolation, and certain fast-evolving genes involved in reproductive isolation can effectively reduce hybrid reproduction. The evidence of fixed differences in genes involved in reproduction and dietary differences supports distinctness, differentiation, diagnosability and divergence expected at the species level of evolutionary trajectory.

## 3. Discussion

Multiple lines of evidence indicate that the resident and Bigg’s ecotypes are genetically distinct, including divergent mitochondrial haplotype lineages with fixed differences, evidence against male-mediated gene flow and diagnosable morphological, as well as mtDNA and nuDNA, differences. Other, less diagnostic types of evidence are also indicative of distinctness and divergence. These include ecological and behavioural (social organization and dispersal, diet, acoustics), morphological and genetic differences (summarized in electronic supplementary material, table S1). These combined lines of evidence, taken together, support divergent evolutionary trajectories consistent with species.

Morphological diagnosability is considered strong evidence of both genetic isolation and ecological specialization consistent with divergence along different evolutionary trajectories [10,71]. While observable differences in colouration patterns, dorsal fin shape and size, and some acoustic characteristics are sufficient for experts to distinguish between resident and Bigg’s individuals or groups in the field and would support subspecies-level divergence, these measures are not diagnostic. However, multivariate analysis results of skull and jaw characteristics, thought to be under selection owing to different dietary specializations (mammals versus fish), are diagnostic (figure 2; e.g. [103]) and strongly support independent evolutionary trajectories or species-level divergence. These morphological differences are further supported by recent data quantifying significant differences in photogrammetrically measured body lengths between sympatric residents and Bigg’s in the eastern North Pacific [20].

Both paternity analyses of contemporary groups [78] and population genetic analyses indicate the absence of male-mediated gene flow in sympatry and the divergence of ecotypes to the point of complete diagnosability (table 1, figures 3 and 4) [13,56,58,59,80,119]. Although the range of both ecotypes in the western Pacific remains uncertain, genetic data from the southeastern extent of their ranges to as far west as the Sea of Okhotsk and south into the Kuril Islands in the western regions indicate genetic cohesiveness of the ecotypes across the range (figures 1 and 3), with mitochondrial haplotypes associated with Bigg’s and resident ecotypes found as far south as Japan [48,54]. The estimates of the timing of the divergence differ for mitochondrial and nuclear genomic data. Time-calibrated mitogenome phylogenies and pseudo-diploid coalescent analysis of nuclear genomes indicate a most recent common ancestor of the lineages approximately 350 000 and 300 000 years ago, respectively [15,59]. The more recent estimate of divergence (~200 000–300 000 years ago) from nuclear genome data has been interpreted to reflect small amounts of post-divergence gene flow, potentially through intermediate populations (e.g. the offshore ecotype; [59]). The period of greatest radiation in the Delphinidae occurred about 2–6 Myr ago [121]. However, there are examples of shorter divergence times among mammalian species (e.g. 43 species pairs with divergence times estimated under 500 000 years; electronic supplementary material, figure S1). The radiation of killer whales globally has occurred within the past ~350 000 years (~14 000 generations) [15,59]. The divergence of ecotypes is common in killer whales and is likely accelerated by the interaction of matrilineal founder events, social organization and dietary specialization. The split between the North Pacific resident and Bigg’s ecotypes suggests an early divergence within the species [7,9,13,15,59].

The guidelines for delineating cetacean subspecies and species with mtDNA (in the absence of other evidence) in Taylor *et al*. [71] suggest a first threshold that subspecies should exhibit at least 95% diagnosability and net nucleotide divergence (*d*_A_) of at least 0.004, and Morin *et al*. [114] suggested the *d*_A_ threshold of 0.0006 for complete mitogenomes. The thresholds for subspecies are met for both control region and mitogenome datasets (table 1), including highly divergent and unshared mitogenome haplotypes with fixed differences (figure 5) [7,15], while the corresponding divergence thresholds for species are not met. Based on simulated data [114,122], the relatively small effective population size for killer whales [13,60,118] is likely to increase both *d*_A_ and diagnosability owing to genetic drift, which could lead to over-classification based on mtDNA alone, but Foote *et al*. [9] argue that these features of killer whale ecotypes may accelerate the speciation process by reducing genomic diversity within ecotypes, and increasing LD and selection, combined with assortative mating to promote reproductive isolation and speciation.

Three recent studies of other cetaceans have proposed taxonomy revisions based on genetic data and other lines of evidence comparable to the data summarized here for resident and Bigg’s ecotypes and also represent relatively recent divergence. For North Atlantic coastal and offshore bottlenose dolphin ecotypes (*Tursiops truncatus*, *T. erebennus*) [123], Indus and Ganges River dolphins (*Platanista minor, P. gangetica*) [104] and two species of finless porpoise (*Neophocaena phocaenoides*, *N. asiaeorientalis*) [72,105], mtDNA control region *d*_A_ was 0.027, 0.0045 and 0.0033, respectively, spanning the guideline thresholds (0.004 for subspecies; 0.02 for species) and divergence times from approximately 500 000 years (*Plantanista*) to ~18 000 years (*Neophocaena*). However, like Bigg’s and resident killer whales, all of these cases also evidenced high diagnosability in mtDNA and morphology, and other supporting lines of evidence that serve as proxies for reproductive isolation and evolutionary divergence are regarded as strong support for species designations [10,71].

### 3.1. Conclusion

Based on the combined lines of evidence presented above, we recommend that resident and Bigg’s killer whales be recognized as species distinct from the globally distributed *O. orca* and from each other. Prior species designations, typically based on a single skull, have all been synonymized under *O. orca*, and there are no prior subspecies designations or holotypes within killer whales of the North Pacific [2]. Three types have been attributed to the ‘North Pacific’, though Gray [124] indicated that he doubted the origin of the specimen for *O. pacifica* [124], and the mitogenome haplotype of this specimen is most similar to Antarctic type B haplotypes [15]. The other two binomials from the Pacific (*Orca ater*, *Orca rectipinna* [125]) are essentially based on visual observation and are not associated with available type specimens. As these two binomials are described in the same publication with no prior names from the North Pacific, we propose *Orcinus ater* and *Orcinus rectipinnus*, for the resident and Bigg’s species, respectively. We discuss the history and use of these binomials in more detail below.

### 3.2. Conservation implications

Many killer whale populations are being negatively impacted by human activities such as overfishing and pollution, and such threats are likely to vary substantially among ecotypes and populations (e.g. [39,88,89,126–133]). Effective management requires the delineation of conservation units within the genus *Orcinus* at appropriate taxonomic levels to facilitate different management strategies for stocks that, in most cases, range across the boundaries between the USA, Canada, Mexico and other nations. In the North Pacific, the resident and Bigg’s ecotypes are considered separate stocks according to the NOAA Marine Mammal Stock Assessment reports [134] and are recognized as separate designatable units by the Committee on the Status of Endangered Wildlife in Canada. Within the resident form, the northern resident and southern resident killer whale (NRKW and SRKW) populations in USA and Canadian Pacific waters are listed as Threatened (NRKW) under the Canadian Species at Risk Act (SARA) and Endangered (SRKW) under SARA and the US Endangered Species Act (ESA), requiring appropriate assessment and management. The Cetacean Specialist Group of the International Union for the Conservation of Nature (IUCN) considers the Society for Marine Mammalogy’s accepted list of taxonomy to assess cetaceans. Currently, the assessment is for *O. orca* globally, and the species is listed as Data Deficient due to taxonomic uncertainty (IUCN Red List, consulted on 3 January 2023). Should the taxonomic change proposed here be accepted by the Society for Marine Mammalogy’s taxonomy committee, two new assessments will result. Such taxonomic refinement, always towards a smaller distribution, has resulted in listing several new taxa in categories of threat (as happened, for example, when the humpback dolphins, *Sousa* spp., were split from two species into four, most with more restricted ranges [135]). The remainder of killer whales globally, including the North Pacific offshore ecotype, will continue (for now) to be classified as *O. orca*, with substantial taxonomic uncertainty remaining.

## 4. Taxonomic treatment and nomenclature

There are three potentially available species names for North Pacific killer whales: *O. ater* [125], *O. rectipinna* (=*rectipinnus*)^2^ [125] and *O. pacifica* (=*pacificus*)^2^ [124]. There appear to be no type specimens available for the first two, though there is a holotype for *pacificus* at the Natural History Museum, London (NHMUK; No. 1165 a). If *rectipinnus* and/or *ater* can be matched with resident or Bigg’s killer whales, then those names dating from 1869 could be used; *pacificus* was not published until a year later (1870) and would only be used if the earlier names are not appropriate and it can be associated with one of the two ecotypes.

The holotype of *O. pacificus* was also originally one of two syntypes used to describe *O. capensis* [136], a species thought to be mainly from the Southern Ocean (the other syntype was from South Africa). Later, Gray [124] considered this skull to be a distinct species and gave it the name *O. pacificus*. Although the collector, Capt. Delville, apparently told Gray that the *O. pacificus* specimen was collected from the North Pacific, later Gray cast doubt on this collection locality [137]. It now seems likely that the skull was actually collected from the coast of Chile, outside the North Pacific, and this appears to be supported by molecular analyses that associate it with Antarctic specimens [15]. The holotype of *O. pacificus* is still present at the NMHUK (No. 1165 a; Jefferson, unpublished data). However, as there is convincing evidence that the specimen came from outside the North Pacific, *O. pacificus* should not be under further consideration as a name for any North Pacific endemic species being described.

**Figure 6.**
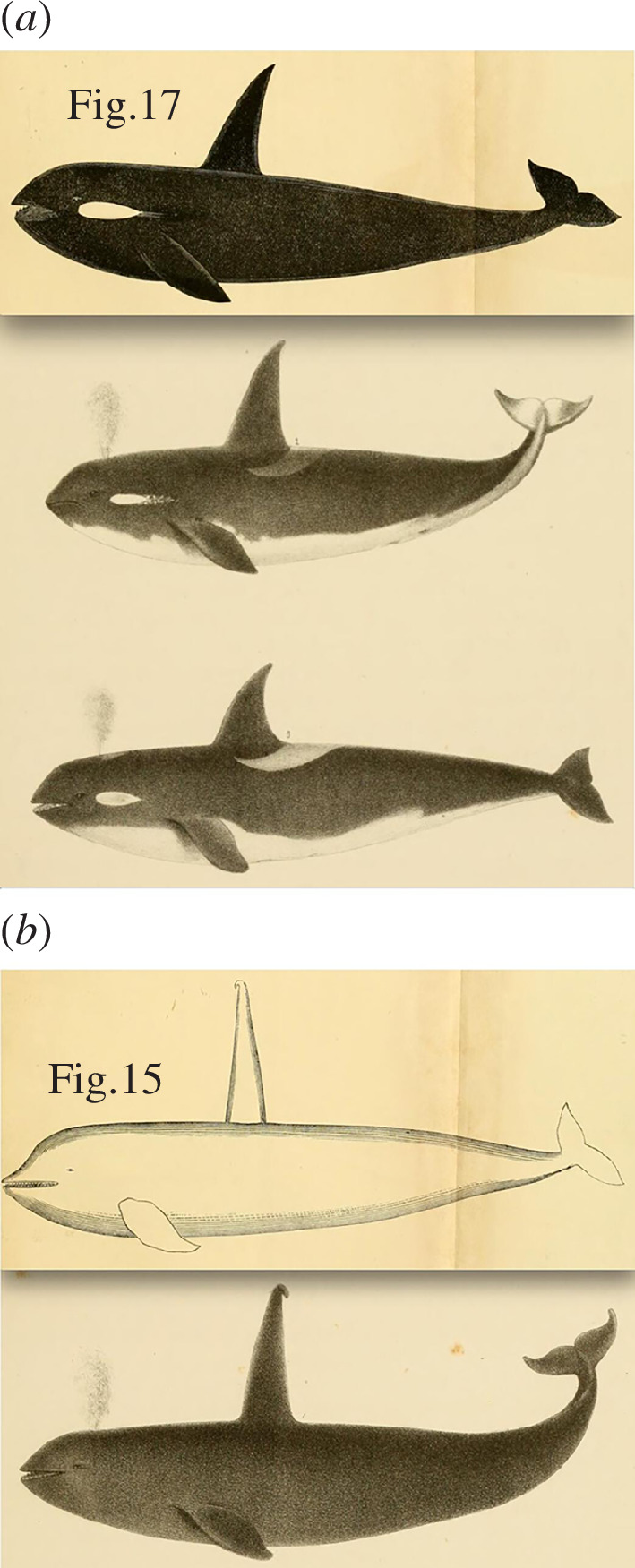
Illustrations of (*a*) *O. ater* and (*b*) *O. rectipinnus* from Scammon [138,140]. These illustrations were likely made by Scammon, or made under his guidance from his field notes and sketches. Whether they represent renderings of specific specimens, or composite sketches, is unknown.

Unfortunately, without type specimens to examine for *rectipinnus* and *ater*, we are left to deal with the descriptions and illustrations (figure 6) provided by Cope (in Scammon [125]) and Scammon [138–140]. These have some relevant details but do not provide clear evidence to associate either ecotype with *rectipinna* or *ater*. The descriptions and illustrations make it clear that Scammon and Cope were not paying attention to the fine details that allow present-day biologists to distinguish the two ecotypes, and they provided no indication that they noticed different prey preferences between them either. So, their split into two species (*rectipinna* and *ater*) was based mostly on features that are not taxonomically informative (e.g. sexually dimorphic dorsal fin sizes/shapes, perceived size differences from sightings at sea, minor colouration differences that are subject to individual variation). It is evident that Scammon, at times, thought that the distinction between the large dorsal fins of male killer whales and the smaller, more falcate fins of females were taxonomically informative. His descriptions and illustrations are mostly the result of observations at sea (figure 6) and show that when the descriptions were written, he only had the opportunity to examine a single specimen in detail and up close (figure 6). This specimen (a 15′ female, designated as *O. rectipinna*) was taken along the California coast, and it contained seals in its stomach ([138], pp. 56–57)^3^.

Several illustrations were provided by Scammon and Cope (i.e. [125,138–140]), but these provide little help, as they do not show accurate details of killer whale colour patterns (e.g. *rectipinna* is shown with a uniform black colour pattern—figure 6) and give no indication of the differences in saddle and dorsal fin shapes that are now known to be reliable indicators of resident versus Bigg’s ecotypes. Scammon considered the largest individuals with the most erect dorsal fins to be of the species *rectipinna*; we now know these are exclusively physically mature adult males. The species *ater* was applied to animals with shorter, more falcate fins, which we now know to be females and younger, ‘sprouting’ males. Scammon obviously did not understand this sexual dimorphism and thought that both sexes were present in both of these ‘species’ (Scammon called them ‘high- and low-finned orcas’). Cope’s descriptions are based strictly on the information provided by Scammon; it is doubtful if Cope (who did not conduct field research at sea) ever saw a North Pacific killer whale, alive or dead. The illustrations in Scammon [138] can be considered as showing ‘proleptic type specimens’ for the two species because it did not become common practice to designate official holotype specimens until the end of the nineteenth century, decades later (see [141]). But, as these specimens are apparently no longer extant, no acceptable name-bearing type specimens currently exist.

That said, the descriptions provided by Cope (in Scammon [125]) and Scammon [138] do provide some information that, while not completely unambiguous, does suggest that the two species, *rectipinnus* and *ater*, can be tentatively allied with the two ecotypes. Based on this information, *ater* would be associated with residents (e.g. more northern range from Oregon to the Aleutians; ‘proleptic type specimen’ from the Strait of Juan de Fuca, where residents are common; no specific remarks on observed prey types) and *rectipinnus* with Bigg’s (e.g. range description from California southwards; ‘type specimen’ from California, where Bigg’s are common and residents are very rare; multiple specific reports of attacks on whales, porpoises and pinnipeds). Again, these are not 100% clear, and there is some conflicting information in the various accounts, but the balance of the evidence suggests that *ater* would be associated with the resident and *rectipinnus* with Bigg’s ecotypes. If the CAS skull collected by Scammon was indeed at the time a type specimen for *rectipinnus* (see fn 3), then the finding of seal remains in its stomach ([138], pp. 56–57) would clearly associate that species with the Bigg’s ecotype.

For the names *rectipinnus* and/or *ater* to be used, it becomes necessary to designate a neotype specimen for each of them, chosen from among existing specimens at institutions that could be unambiguously identified with the two ecotypes. The designation of neotype specimens for the two proposed species below removes any uncertainty about the assignment of the names *rectipinnus* and *ater,* and forever unambiguously links these two names to the corresponding ecotypes (i.e. Bigg’s and resident killer whales).

### Species redescriptions

Order Artiodactyla Montgelard, Catzefils and Douzery, 1997

Cetacea Brisson, 1762

Odontoceti Flower, 1867

Superfamily Delphinoidea Flower, 1865

Family Delphinidae Gray, 1821

### *Orcinus rectipinnus* (Cope in Scammon, 1869)

#### Etymology

In Latin, *recti* means right or upright, and *pinna* means fin, feather or wing, most likely referring to the tall, erect dorsal fin of males.

#### Synonymy

*Orca rectipinna* Cope in Scammon, 1869: 22; original designation.

#### Common name

We propose continued use of the common name, ‘Bigg’s killer whale’, for this species, to honour Dr. Michael A. Bigg (1939–1990), who pioneered the study of North Pacific killer whales in the 1970s. This ecotype was formerly known as the ‘transient killer whale’.

#### Type specimen

USNM 594671

**Figure 7.**
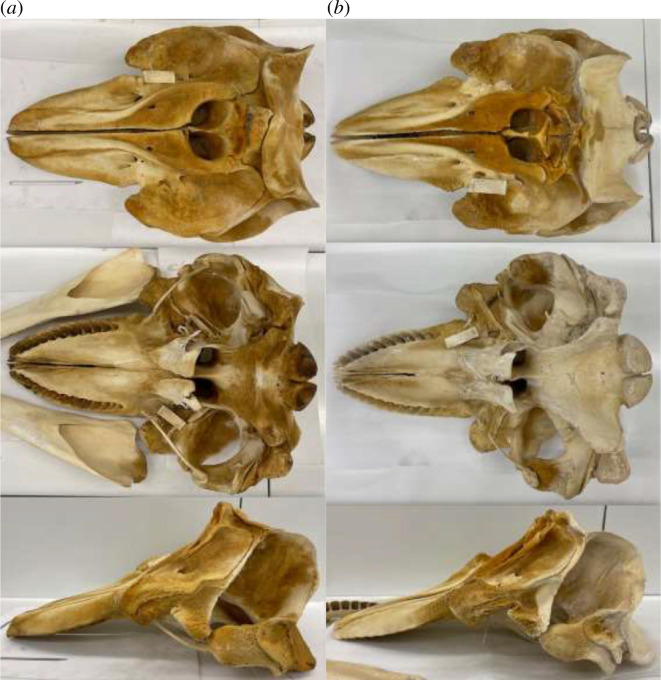
Photographs of neotype skulls for (*a*) *Orcinus rectipinnus* (USNM 594671) and (*b*) *Orcinus ater* (USNM 594672).

No type specimen is extant from the original description (Cope in Scammon [125]), so we have designated a neotype. The neotype is the skull of a physically mature male (total length 731 cm, CBL of cranium 1124 mm) in the U.S. National Museum of Natural History Marine Mammal Collection, deposited under museum number USNM 594671. The skull was previously in the NOAA National Marine Mammal Laboratory collection as NMML 0082. It is illustrated in figure 7*a*. mtDNA control region haplotype (160 bp) ‘T’ (SWFSC ID 39064 in 142]) unambiguously identifies this specimen as a Bigg’s killer whale. Morphological analysis of this specimen was included in Fung [103]. Detailed measurements of the type specimen are in electronic supplementary material, table S2.

#### Type locality

The neotype was collected by J.E. Eckberg on 22 September 1966, near San Francisco, CA, USA.

#### Diagnosis

Bigg’s killer whales differ from residents in growing to somewhat larger sizes, and having a wider-based, more triangular dorsal fin that is more pointed at the tip. The dorsal fin also tends to be less falcate (even in females). The saddle patch behind the dorsal fin extends further forward than it does in residents, usually to well past the mid-point of the dorsal fin base, and may appear larger compared to that of residents [40,79]. Virtually all saddle patches are closed (with no significant invasion of black), and many are rounded, with their forward extensions not ending in a point. These correspond to the ‘smooth’ or ‘bump’ patch types of Baird and Stacey [22].

Bigg’s killer whales have longer and more robust skulls than residents, with the following measurements showing significantly greater average values: condylobasal length, postorbital width, occipital width, width of rostrum at base and length of mandible [103]. While individual measures show modal differences with some overlap, canonical variate analysis of both cranial measures and measures of mandibular morphology yields distinct clusters for the two ecotypes ([103]; figure 2). Genetic analyses of mtDNA are diagnostic, based on fixed sequence differences (table 1), while nuDNA allele frequency differences allow diagnosis based on cumulative assignment probability (e.g. assignment tests or PCA; figures 3 and 4).

#### Description

**Figure 8.**
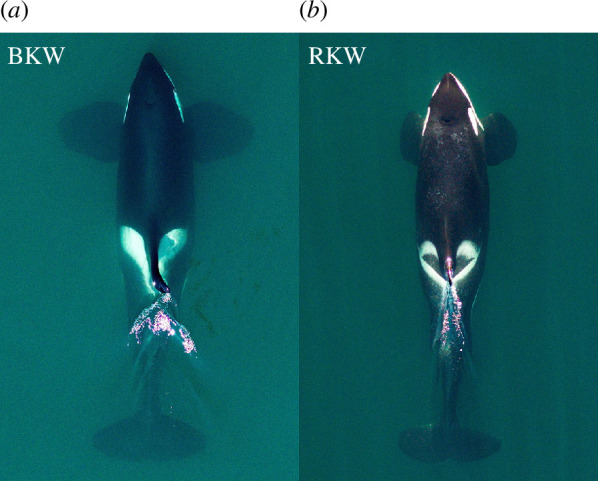
Vertical images of (*a*) an adult male Bigg’s killer whale (BKW) from the West Coast Transient population of Bigg’s killer whales and (*b*) an adult male resident killer whale (RKW) from the sympatric Southern Resident population of resident killer whales. Images are scaled to the estimated asymptotic lengths of 7.3 m [20] and 6.9 m [145], respectively. Vertical images were collected using an octocopter drone using methods described by Durban *et al*. [146], provided by John Durban and Holly Fearnbach.

This is a species of killer whale, reaching total lengths of at least 830 cm in males and 710 cm in females [20] and weights of 6600 kg in males and 4700 kg in females [143]. It has the basic features of the killer whale body plan: a robust body with a tall dorsal fin near the centre of the back, large paddle-shaped flippers, broad flukes with a slightly convex trailing edge and a blunt head with a short, poorly defined beak (figure 8*a*). There is sexual dimorphism, with males growing much larger than females, and near sexual maturity developing a tall (up to at least 1.5 m), erect dorsal fin and much larger flukes and pectoral fins. The mouthline is straight, with a small downturn at the gape. The basic killer whale colour pattern is largely dark grey to black, with a white ventral field that has lobes extending up and back along the tail stock, a white post-ocular patch, and a light grey to white ‘saddle patch’ behind the dorsal fin. The lower jaw and the undersides of the flukes are mostly white, but the entire flippers and dorsal fin are black. The areas of light and dark are generally well defined, with a crisp border. Each tooth row of both upper and lower jaws contains 10–14 large, conical teeth [144].

#### Comparison to other taxa

Bigg’s killer whale is one of three proposed species of killer whales (genus *Orcinus*) globally. It is endemic to the North Pacific Ocean and adjacent seas, and it preys primarily on marine mammals (as opposed to bony fish and elasmobranchs). It can be distinguished from other killer whale species and ecotypes by its genetic profile, its morphology and colouration (see *Diagnosis* above), and also acoustically.

#### Distribution

Bigg’s killer whales occur throughout the waters of the eastern North Pacific Ocean from at least northern Baja California, Mexico, through to eastern Russia and northern Japan in the western Pacific [48,147,148]. Their distribution extends to the Okhotsk Sea and Arctic Ocean (e.g. Chukchi Sea; figure 1). While they are most commonly observed over the continental shelf and in inshore waters, they may also occur in oceanic waters beyond the continental shelf edge [147].

### *Orcinus ater* (Cope in Scammon, 1869)

#### Etymology

In Latin, *ater* means black or dark, which probably refers to the largely black colour of this species.

#### Synonymy

*Orca ater* Cope in Scammon, 1869: 22; original designation.

#### Common name

We are planning on engaging with North American Indigenous tribal groups and expect to eventually have a consensus common name, but in the meantime, we suggest continued use of ‘resident killer whale’ so as to maintain consistency.

#### Type specimen

USNM 594672

No type specimen was preserved from the original description [125], so we have designated a neotype. The neotype is a physically mature male (total length 698 cm) in the U.S. National Museum of Natural History Marine Mammal Collection. The skull (CBL 1019 mm) is deposited under museum number USNM 594672 and was collected by C.H. Fiscus, H. Kajimura and M. Keyes on 28 February 1967. The skull was previously in the NOAA National Marine Mammal Laboratory collection as NMML 0089. It is illustrated in figure 7*b*. mtDNA control region haplotype (160 bp) ‘SR’ [SWFSC ID 39071 in 142] unambiguously identifies this specimen as a resident killer whale. Skeletal morphological analysis of this specimen was included in Fung [103]. Detailed measurements of the type specimen are in electronic supplementary material, table S2.

#### Type locality

The neotype specimen was collected in Yukon Harbor, Puget Sound, Washington, USA (lat: 47.53, lon: −122.52).

#### Diagnosis

Resident killer whales are slightly smaller than Bigg’s, with total lengths not exceeding 725 cm in a moderate sample [103,143,145]. They have a shorter-based, more falcate dorsal fin that tends to be more rounded at the tip. The post-dorsal fin saddle patch generally does not extend forward much past the mid-point of the dorsal fin base. Individuals show a variety of saddle patch types, including closed (with no invasion of black), open (with a large invasion of black extending from above) and cupped (with a small ‘scoop’ or notch of black invading the saddle from above; e.g. [40, 79]). These correspond to all five patch types of Baird and Stacey [22].

Resident killer whales have smaller and more gracile skulls than Bigg’s, with the following measurements showing significantly lower average values: condylobasal length, postorbital width, occipital width, width of rostrum at base and length of mandible [103]. While individual measures show modal differences with some overlap, PCA of both cranial and mandibular morphology resulted in distinct clusters for Bigg’s and resident species ([103]; figure 2). Genetic analyses of mtDNA are diagnostic, based on fixed sequence differences (table 1), while nuDNA allele frequency differences allow diagnosis based on cumulative assignment probability (e.g. assignment tests or PCA; figures 3 and 4).

#### Description

This is a species of killer whale, reaching total lengths of at least 725 cm in males and 644 cm in females [145]. The basic killer whale body plan includes a robust, spindle-shaped body, with a tall dorsal fin positioned at the centre of the back, large paddle-shaped pectoral fins, wide flukes with a slightly convex trailing edge and a blunt head (figure 8*b*). There is a short, poorly defined beak. This species shows extreme sexual dimorphism, with males growing much longer and heavier than females; males also develop a tall (up to 2.25 m [149,150]) erect dorsal fin that may actually cant forward (making it look like it was put on backwards), and much larger flukes and pectoral fins. There is a straight mouthline, ending in a slight downturn at the gape. Resident killer whales show the basic colour pattern found in all members of the genus: most of the body is dark grey to black, with a white ventral field that branches into lobes extending up and back along the caudal peduncle. There are large white post-ocular patches, and a light grey to white ‘saddle patch’ originating behind the dorsal fin. The undersides of the flukes are mostly white, as is the entire lower jaw, but the flippers and dorsal fin are black on both sides. There are 10–14 large, conical teeth in each tooth row [144].

#### Comparison to other taxa

The resident killer whale is one of three proposed species of killer whales (genus *Orcinus*) globally. It is endemic to the North Pacific Ocean and adjacent seas, and it preys primarily on bony fish, especially North Pacific salmon *Oncorhynchus* spp. (as opposed to marine mammals and elasmobranchs). It can be distinguished from other killer whale species and ecotypes by its genetic profile, its morphology and colouration (see *Diagnosis* above), and also acoustically.

#### Distribution

Resident killer whales occur in inshore and continental shelf waters, primarily from Oregon in the eastern Pacific to eastern Russia and northern Japan in the western Pacific [48,54,147,148,151]. Pods occasionally move southwards down the U.S. west coast to as far as Monterey Bay, CA [152]. They also inhabit the Okhotsk Sea and the southern Bering Sea (figure 1).

## Data Availability

No new data were generated in support of this research. Methods for principal components reanalysis of genetic data are presented in supplemental materials. Genotype data that were from previous studies [56,113], but not publicly available, have been archived in the Dryad Digital Repository [154]. Electronic supplementary material is available online [155].

## Footnotes

^1^
An argument for sufficiency should weigh the evidence from genetics, morphology and behaviour leading to the conclusion that lineages have diverged and can no longer converge.

^2^
The -*us* ending is required for Latin masculine gender agreement with the genus *Orcinus*.

^3^
This is probably the same specimen associated with a skull reported by Dall (1874) from the California Academy of Sciences (CAS). If so, this skull collected by Scammon would have been the holotype specimen for *O. rectipinnus*. However, any such specimen is no longer in existence, as the 1906 San Francisco earthquake and resulting fire destroyed the entire CAS collection, including all records (M. Flannery, in litt., 8 April 2022).
